# A Method for Isolation and Proteomic Analysis of Outer Membrane Vesicles from Fecal Samples by LC-MS/MS

**DOI:** 10.4172/0974-276X.1000494

**Published:** 2019-03-18

**Authors:** Jing Wu, Mingrui An, Jianhui Zhu, Zhijing Tan, Grace Y. Chen, Ryan W Stidham, David M. Lubman

**Affiliations:** 1 Department of Surgery, University of Michigan Medical Center, Ann Arbor, Michigan 48109, USA; 2 Department of Internal Medicine, Division of Hematology/Oncology University of Michigan, Ann Arbor, MI 48109, USA; 3 Department of Internal Medicine, Division of Gastroenterology and Hepatology, University of Michigan, Ann Arbor, MI, 48109, USA; 4 Department of Microbiology and Immunology, University of Michigan, Ann Arbor, MI 48109, USA

**Keywords:** Outer membrane vesicles, Bacteria, Fecal samples, Ultracentrifugation, Filter

## Abstract

Outer membrane vesicles (OMVs) are nanosized spheres secreted by bacteria that are similar to the vesicles known as exosomes, which are secreted by most mammalian cell types. In contrast to many studies focusing on optimizing methods for enriching exosomes from biological fluid, few studies have been conducted to investigate outer membrane vesicles from fecal samples. Herein, we have developed a pipeline comprised of membrane filtration and multiple cycles of ultracentrifugation (UC) to isolate OMVs from fecal samples for proteomics analysis, where multiple cycles of UC are required for removal of contaminants. By iTRAQ labeling quantitative proteomics analysis, different filter sizes (0.22 μm and 0.45 μm) were compared in terms of their performance in enriching OMVs and eliminating background fecal material. Using the 0.45 μm filter, a slightly higher protein yield was obtained but no additional contaminating proteins from bacteria were identified compared to those from the 0.22 μm filter. The 0.45 μm filter together with the multiple cycles of UC were thus used to isolate OMVs for proteomics analysis. To our knowledge, this is the first study profiling a large number of OMV proteins from fecal samples. Such capabilities may help provide valuable information in understanding the communication between the host and microbiota, which is critical in preventing cancer and disease development

## Introduction

Gut microbiota have the central role in maintaining intestinal homeostasis and the well-being of the host, which is regulated by communication between the host and microbiota. Recently, bacterial outer membrane vesicles (OMVs) have been found to be the key player in bacteria-host communication [1]. OMVs are typically 20 to 300 nm nanostructures that are produced by most Gram-negative bacteria. OMVs produced by Gram-positive bacteria have been implicated in the pathogenesis of many diseases [2]. Moreover, the ability of OMVs to transfer proteins, lipids and nucleic acids to tissues, affords potential application in therapy delivery.

**Figure 1:**
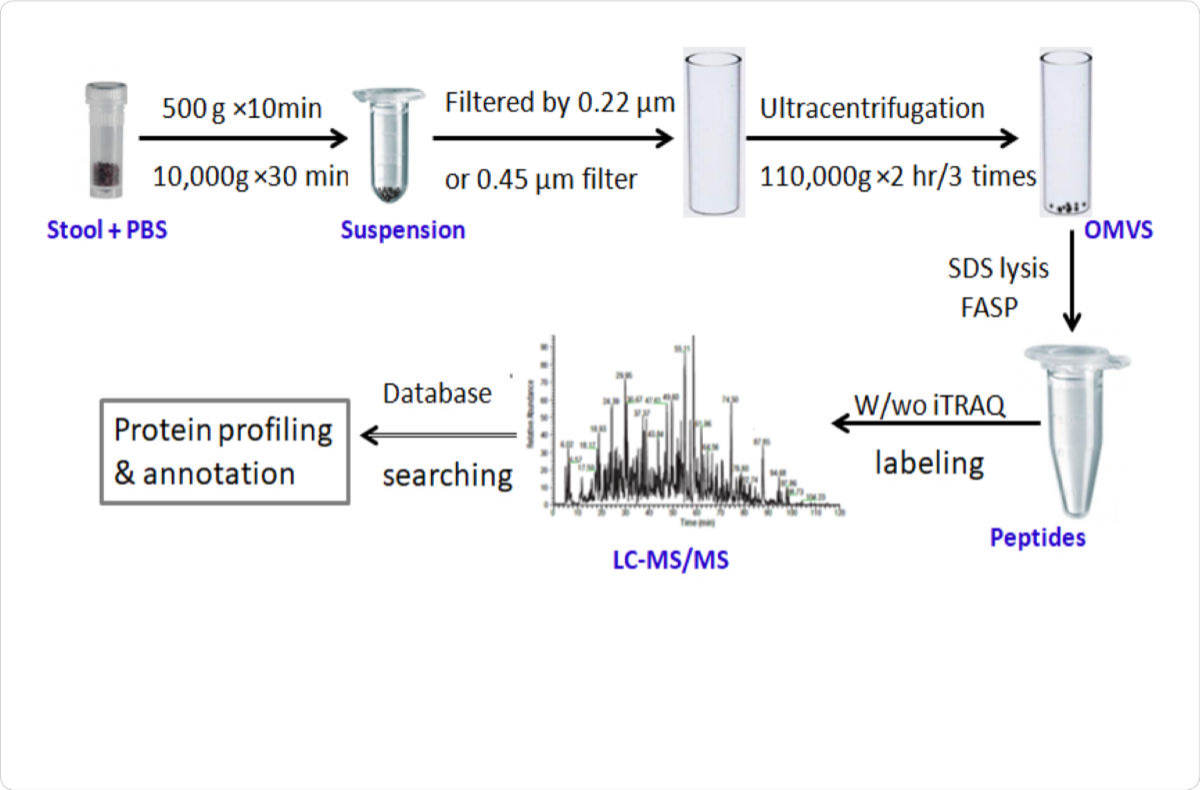
The workflow of OMVs isolated from stool samples. This involved a pipeline comprised of membrane filtration and multiple cycles of ultracentrifugation (UC) to isolate OMVs from fecal samples for proteomics by LC-MS/MS.

Interaction between bacterial OMVs and the host modulates development and function of the immune system, which plays a critical role in disease and cancer development. Kim et al. [3] reported that bacterial OMVs are capable of inducing long-term antitumor immune responses within the tumor microenvironment. Emerging evidence has shown that dysbiosis of gut microbiome is closely related to the development of Crohn’s disease, one type of inflammatory bowel disease (IBD) [4]. OMVs secreted by the human commensal *Bacteroides fragilis* showed the ability to protect from colitis by inducing an anti-inflammatory cytokine (IL-10) production [5]. Several studies have shown that bacterial RNA contained within OMVs were involved in modulating the host immune responses [6–8]. The identification of specific protein components within OMVs may have great value for understanding the mechanism by which the OMVs interact with and regulate the innate immune responses in the host. Isolation of bacterial OMVs from culture medium has been reported [9–11], but bacterial OMVs from fecal samples may provide better insight into the functioning of the gut microbial community. Herein, we present an integrated methodology for enriching OMVs from human and mouse stool samples for identification of proteins carried in these bacterial OMVs. Multiple cycles of ultracentrifugation were performed to pellet OMVs and remove contaminants. Different size filters (0.22 μm and 0.45 μm) were compared for sample processing in terms of the performance in reducing contamination from bacteria and the improvement in OMV protein identification. On the basis of these comparisons, we developed an integrated pipeline to process fecal samples for OMV proteomic analysis (Figure 1). Such methodology is needed for future investigations of OMVs function in immune regulation and their roles in pathogenesis.

## Materials and Methods

### Fecal samples

Germ-free mice (GF) were gavaged with a defined community of 11 bacterial strains including *Bacteroides vulgatus, Bacteroides uniformis, Bacteroides thetaiotaomicron, Escherichia coli, Barnesiella intestinihominis, Parabacteroides distasonis, Faecalibacterium prausnitzii, Bifidobacterium longum, Eubacterium rectale, Lactobacillus reuteri, and Roseburia inulinivorans* [12]. Fecal samples were collected from mice and immediately stored at −80°C. Colonization by all 11 members was confirmed by isolating bacterial DNA using the DNeasy Powersoil Kit (Qiagen) followed by qPCR using bacterial-specific primers at one and four weeks post-gavage. Animal experiments used protocols approved by the University Committee on Use and Care of Animals. Human stool samples were prospectively collected from patients with Crohn’s disease at the University of Michigan. IBD diagnoses required prior endoscopic and histologic evidence supporting either ulcerative colitis or Crohn’s disease with diagnostic agreement from the treating gastroenterologist. Written informed consent for each subject was obtained following a protocol approved by the University Review Board. Patient’s stool samples were collected using gloves, a tongue depressor, collection hat and transfer into a sterile container. Selected patients had not been exposed to antibiotics or probiotics for 1 year prior to stool collection and were on stable medical therapy (no changes for a minimum of 3 months). Collected stool was immediately refrigerated and frozen at −80°C within 12 hours of production. For the purpose of developing the methods herein 1 each of human and mouse stool samples were used.

### Isolation of outer membrane vesicles (OMVs) by multiple cycles of ultracentrifugation

Fecal samples (150 mg human stool and 25 mg mouse stool) were thoroughly suspended in 500 μL PBS by extensive vortex. Disrupted pellets were centrifuged at 500 × g for 10 min to precipitate insoluble materials, to which 500 μL PBS was added to re-suspend the pellets. This step was repeated four times. The suspension was then combined and centrifuged at 10,000 g for 30 min to pellet bacteria. This nominally bacteria-free supernatant was further filtered through 0.45 μm or 0.22 μm filters, respectively, to further remove any bacteria. The OMVs were pelleted by ultracentrifugation at 110, 000 g for 2 hr at 4°C in a Beckman Optima XL-70 (Beckman Coulter, Indianapolis, IN). After removing the supernatant, OMVs were suspended in 4 mL PBS and ultracentrifuged at 110,000 g for 70 min at 4 °C. This step was repeated three times where multiple cycles of ultracentrifugation were used to remove possible contaminants as shown in prior work [13,14].

### Transmission electron microscopy (TEM)

The morphology of isolated OMVs was evaluated by TEM as described previously [13,14]. Briefly, 5 μl of each of the OMV samples was added to a carbon film and incubated for 2 min. After removing the supernatant liquid by filter paper, 5 μL of 2.5% w/v glutaraldehyde in PBS were added to fix OMVs onto the carbon film. After washing the carbon film three times, the OMVs were negatively stained with 5 μL of 1% uranyl acetate for 1 min, then imaged with a Philips CM-100 TEM instrument.

### Protein extraction, trypsin digestion and iTRAQ labeling

The isolated OMVs were lysed with 2% SDS buffer at 95°C for 10 min. The suspension was centrifuged at 13,000 rpm for 30 min to collect the lysates, whose soluble protein concentration was measured by the BCA protein assay (Thermo Scientific, Waltham, MA). Twenty micrograms of protein extract were reduced by 25 mM TCEP at 56 °C for 1 h. The concentrates were then diluted in 200 μL of 8M urea in 50 mM TEAB solution, followed by alkylation with 50 mM IAA in the dark for 20 min. The concentrates were transferred to Microcon Ultracel YM-30 filtration devices (Millipore, Billerica, MA) and centrifuged at 14,000 g for 15 min. Filters were subsequently washed twice with 8M urea, followed by three washes with 50 mM TEAB. Enzymatic digestion was performed by adding 400 ng of trypsin (Promega, Madison, MI) in 75 μL of 50 mM TEAB to the filter (enzyme to protein ratio 1:50) and incubated overnight at 37°C. Released peptides were collected by centrifugation and desalted with C18 spin columns (Thermo Scientific), followed by dryness using a SpeedVac concentrator (Thermo Savant, Milford, MA). The resulting tryptic peptides were labeled by iTRAQ 4-plex reagent according to the instructions [15,16]. The iTRAQ tags were used so that we could obtain a quantitative comparison between the results of using the 0.22 μm versus the 0.45 μm filter in one run. The labeled samples were mixed, and desalted using C18 spin columns. The eluted samples were dried by SpeedVac prior to mass spectrometry analysis. The iTRAQ labeled samples were analyzed by Orbitrap mass spectrometry in duplicate.

### LC-MS/MS and data analysis

One microgram of peptides was separated on an Easy 1000 nano UHPLC system (Thermo) equipped with a C18 column (Acclaim PepMap RSLC, 75 μm × 25 cm, 2 μm, 100 Å) at a flow rate of 300 nL/min using a 60 min gradient from 2 to 35% acetonitrile in 0.1% formic acid. The eluted peptides were analyzed by an Orbitrap Fusion Tribrid Mass Spectrometer (Thermo Scientific) operated in positive ion mode. MS1 spectra (from *m/z* 375–2000) were acquired in the Orbitrap analyzer with resolution r = 60 000 at m/z 200, and the top 10 most intense ions were selected for tandem MS analysis by collision-induced dissociation (CID) in the linear ion trap. The normalized collision energy was set to 35. Dynamic exclusion was enabled, with a mass exclusion width of 10 ppm and exclusion duration of 20 s. In addition, the samples were also run using the spectral count method without the iTRAQ labels to determine the maximum number of OMV proteins detected. This procedure was performed in quadruplicate. The raw data were searched against the protein database using SEQUEST incorporated into Proteome Discover 2.1 (Thermo Scientific). The protein sequences for human, mouse, and food were downloaded from the Universal Protein Resource Knowledgebase (UniProtKB, released 2014_5). The protein sequences for bacteria in mice were downloaded from the JGI IMG database (https://img.jgi.doe.gov/cgi-bin/pub/main.cgi). For bacteria from human samples, the protein sequences were downloaded from the human microbiome project (https://www.hmpdacc.org/hmp/HMRGD/). The combined host, food, and bacterial protein database was constructed by appending the human/mouse and food (wheat, corn, soybean, yeast, alfalfa) proteins to those of microbes in mouse or human stools. The search parameters were set as follows: precursor ion m/z tolerance, ± 10 ppm; fragment ion m/z tolerance, ± 0.05 Da; two missed cleavages allowed; static modification, carbamidomethylation (+57.02146 Da, C), 4-plex iTRAQ (N-term and K); dynamic modifications, oxidation (+15.99492 Da, M). Identified peptides were filtered using a 1% peptide-level false discovery rate (FDR).

## Results and Discussion

OMVs are small spherical structures enclosed with a membrane bilayer typically ranging between 20 to 300 nm in size. To avoid any bacteria or cell debris contamination, the suspension of feces should be filtered with a pore size filter prior to UC. Due to the upper limit size of around 300 nm, it has been reported that processing the samples with a 0.22 μm filter may lead to a low yield of OMVs that are captured by the filters, while using a 0.45 μm filter may result in contamination from the bacteria. Therefore, in this study, we compared two different sizes of filters (0.22 μm vs. 0.45 μm) in terms of their performance in OMVs enrichment and contaminant removal.

### Size distribution of OMVs

**Figure 2:**
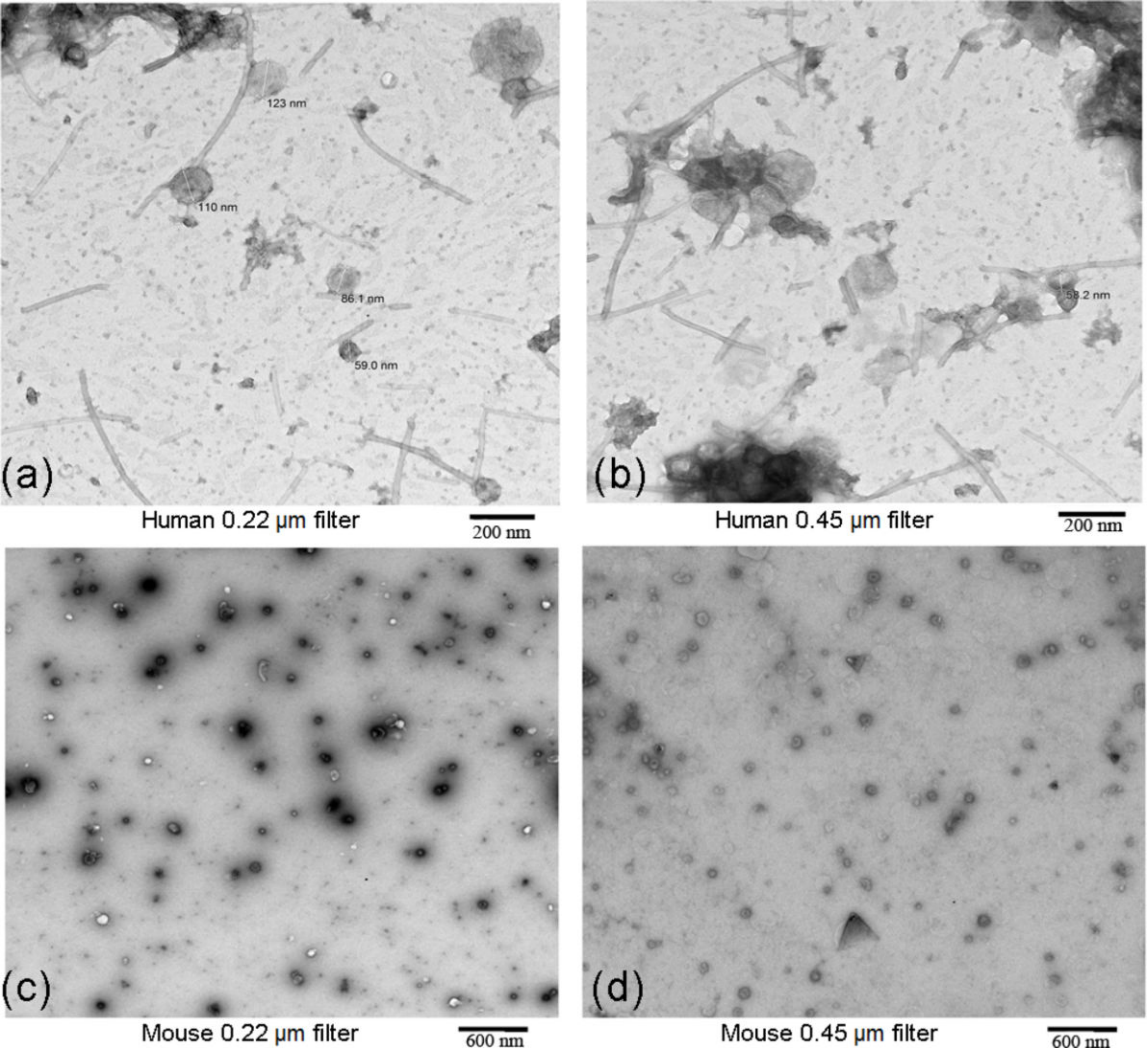
TEM images of OMVs enriched from human and mouse stools processed with the 0.22 μm (2a for humans and 2c for mouse) and 0.45 μm filters (2b for humans and 2d for mouse).

**Figure 3:**
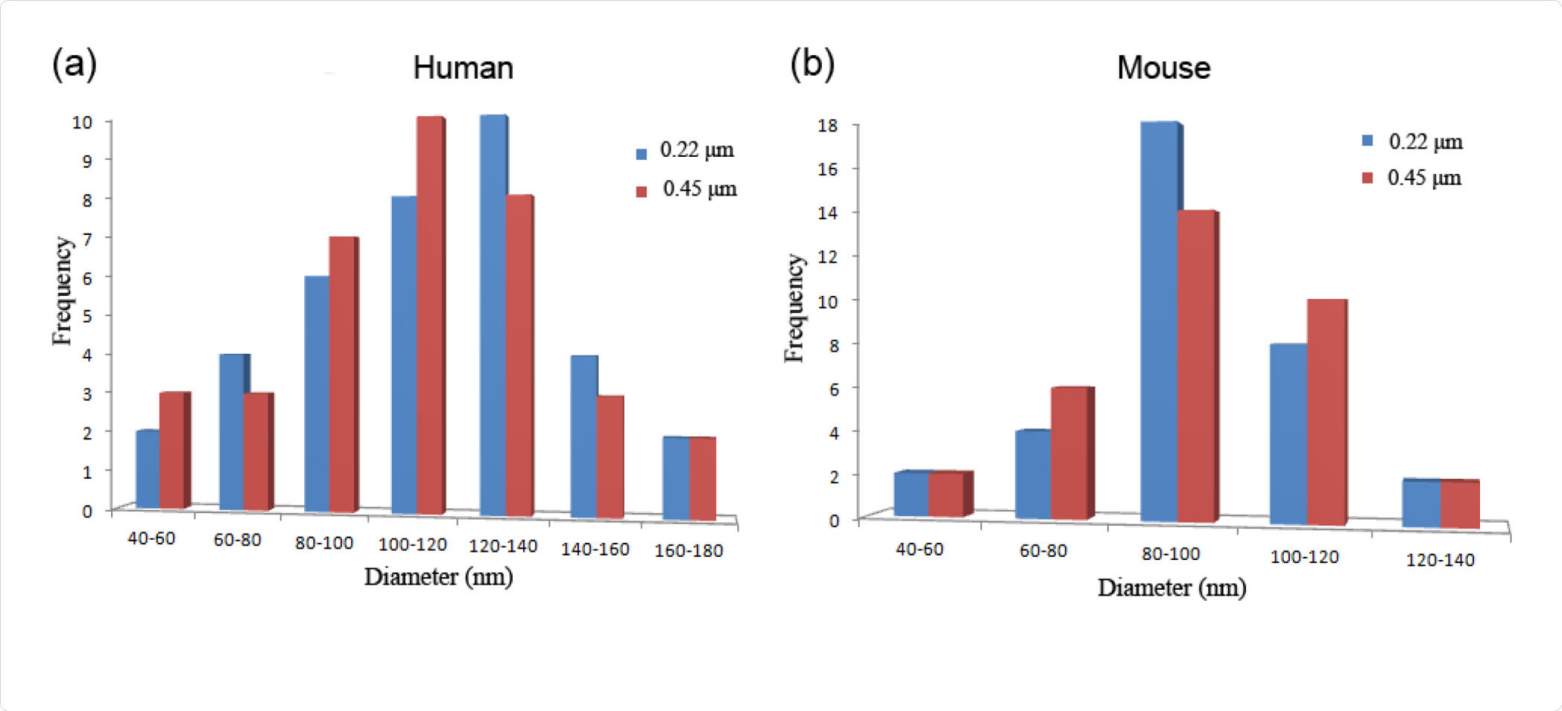
The size distribution obtained by TEM of the outer membrane vesicles extracted from the human and mouse stool samples processed with 0.22 μm and 45 μm filters for humans (figure 3a) and mouse(figure 3b).

In Figure 2, TEM images of OMVs enriched from human (Figure 2a and 2b) and mouse stools are shown (Figure 2c and 2d). In the images obtained from mouse stool, it should be noted that one cannot distinguish between mouse-derived extracellular vesicles versus bacterial OMVs. Also, in the human samples there are tubules observed extending from the OMVs; this has been reported in recent work, although their function is not known. In Figure 3, the corresponding size distribution of OMVs enriched from human (Figure 3a) and mouse (Figure 3b) stool samples using 0.22 μm and 0.45 μm filters as obtained by TEM is shown. The size distributions from 0.22 μm and 0.45 μm filters were found to be similar where the average diameters of OMVs from mouse stools were 78 (± 22) nm and 76 (± 23) nm, respectively, and from human stools were 113 (± 30) nm and 110 (± 29) nm, respectively.

### Protein yield of OMVs

To determine whether the use of 0.22 μm filters could lead to significant loss of OMVs, we measured the protein yield of OMVs using these two different filter sizes based on the BCA assay. The amount of OMV proteins obtained from 150 mg of human stool was 73.1 (± 1.3) μg with the 0.22 μm filter and 76.1 (± 1.4) μg with the 0.45 μm filter, while from 25 mg of mouse stool, the amount of OMV proteins isolated was 12.6 (± 0.8) μg with the 0.22 μm filter, and 14.2 (± 0.3) μg with the 0.45 μm filter. A slightly higher protein yield was achieved using the 0.45 μm filter as compared to that of the 0.22 μm filter but the difference was not significant. However, it should be noted that the 0.22 μm filters tend to clog more than the 0.45 μm filters, which may lead to potential sample loss.

### Protein identification by LC-MS/MS

The OMV proteins processed with different sizes of filters and multiple cycles of UC were labeled with iTRAQ reagents for comparison of protein identification. The reporter ions could be identified if the protein was present in the sample and vice versa. Therefore, by iTRAQ labeling assays, we could quantitatively compare the number of proteins presented in the samples using these two different sizes of filters.

From human stool samples, we identified 376 proteins labeled with iTRAQ from the OMVs processed with both 0.22 μm and 0.45 μm filters, including 87 human proteins from EVs and 289 bacterial proteins from OMVs. Quantitative proteomics analysis of OMVs isolated from human stools is shown in Supplemental Table S1. In the mouse stool samples, 626 proteins were identified from the iTRAQ labeling samples, including 363 proteins from mouse EVs and 263 proteins from bacterial OMVs (Supplemental Table S2). No difference in protein number was observed from samples processed with the 0.22 μm or 0.45 μm filters. These results indicated that the processing of mouse and human stool samples with either the 0.22 μm or 0.45 μm filters produced no significant differences in protein identification. In addition, two extracellular vesicle markers, CD63 and CD82, were identified from the mouse stool samples based on mass spectrometric analysis, and their abundance ratios between filter sizes were 1.5 for CD63 and 0.8 for CD82. This result indicates that there are no significant differences between using the two different sizes of filters in this workflow, where similar performance of these two filters in enriching OMVs was achieved. To evaluate whether using the 0.45 μm filter can lead to bacterial contamination during processing, we processed fecal samples from nominally germfree mice using the 0.45 μm filter. We identified 322 mouse proteins and no bacterial proteins, indicating the absence of bacterial contamination when samples were processed with the 0.45 μm filters.

**Figure 4:**
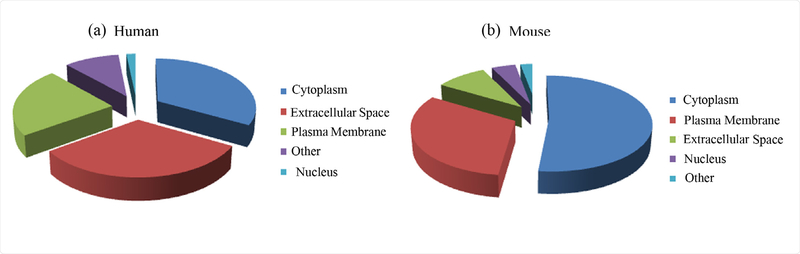
The subcellular distribution obtained from proteomic analysis for (a) human stool and (b) mouse stool which shows that a large number of proteins identified from human and mouse stool samples are from plasma membrane and extracellular space.

In order to profile proteins from stool-derived OMVs, we identified proteins without isobaric labeling. From four replicate MS runs, 746 OMV proteins (623 bacterial proteins and 123 human proteins) were identified from the OMVs enriched from human stools. These bacterial proteins were from more than 95 different bacterial species, more than half of which are Bacteroides including *Bacteroides caccae, Bacteroides cellulosilyticus, Bacteroides dorei, Bacteroides ovatus, Bacteroides uniformis, Bacteroides vulgatus, and Bacteroides xylanisolvens.* All of the proteins identified from OMVs enriched from human stools are listed in Supplemental Table S3. From mouse stools inoculated with the 11-bacteria, we identified 944 OMV proteins (531 mouse proteins and 413 bacterial proteins) (Supplemental Table S4). Ingenuity Pathway Analysis (IPA) was performed to determine cellular locations and functions ofthe identified proteins. OMV proteins from the human stool samples from the cytoplasm, extracellular space, and plasma membrane account for 33.1, 32.2, and 22.9% of the total protein, respectively (Figure 4a), which were mainly involved in antimicrobial response, inflammatory response, and organismal injury and abnormalities. These OMV proteins may vary with the degree of intestinal inflammation and Crohn’s disease phenotype. The impact of OMVs on the pathogenesis of IBD will be examined further in future work as the sample size and disease activity variance was not examined in this feasibility study. OMV proteins from the mouse samples derived from the cytoplasm, plasma membrane, and extracellular space accounted for 51.9, 31.7, and 9.2% of the total protein, respectively (Figure 4b), and these proteins correlated with gastrointestinal disease, inflammatory response and immunological disease. Since the majority of the bacterial proteins lack location and functional information, pathway analysis related to these bacterial proteins could not be performed.

## Conclusion

An integrated pipeline was developed to enrich OMVs from human and mouse stool samples. Two different filter sizes (i.e. 0.45 μm vs. 0.22 μm) were compared in terms of their performance in enriching OMVs and eliminating background fecal material. Based on the BCA and a quantitative mass spec-based assay, a higher protein yield with removal of bacteria was achieved with the 0.45 pm filter compared to that with 0.22 μm filter, and therefore, the larger 0.45 μm filter together with multiple cycles of ultracentrifugation appears optimal for OMV protein extraction from stool samples. With this strategy, 746 and 944 proteins were identified from human and mouse fecal samples derived from OMVs and EVs. Surprisingly, these proteins were principally involved in microbial defense and inflammatory response. To our knowledge, this is the first study profiling OMV proteins from fecal samples where we have developed the appropriate methodology for their isolation and proteomic analysis. There are various procedures that could be implemented in further work to improve on current results. The method could be further improved with regard to purity by using methods such as density gradient ultracentrifugation [17] to eliminate any unwanted contaminants after the procedures described herein. Also, methods such as negative stain EM could be used to distinguish human or mouse EVs from OMVs in the TEM procedures. In addition, flow cytometry might also be used as a final step to separate out human or mouse EVs from bacterial OMVs. Future prospects for this work will be in its applications to biological problems. For example, our results could provide invaluable information in understanding the communication mechanism between the host and bacteria, which plays a critical role in gut health and preventing disease.

## Supplementary Material

Suppl Table 3

Suppl Table 4

Suppl Titles

Suppl table 1

suppl table 2
