# Supplementary material for: A Method for Isolation and Proteomic Analysis of Outer Membrane Vesicles from Fecal Samples by LC-MS/MS
Source: J Proteomics Bioinform. Author manuscript; Available in PMC 2020 Mar 18. (PMC6497448; doi:10.4172/0974-276X.1000494)
Supplement: Suppl Titles [file NIHMS1020350-supplement-Suppl_Titles.docx]

**Supporting Information Available:** Quantitative proteomics analysis of OMVs isolated from human stools is shown in Supplemental Table S1. Quantitative proteomics analysis of OMVs isolated from mouse stools is shown in Supplemental Table S2. All of the proteins identified from the OMVs enriched from human stools are listed in Supplemental Table S3. All of the proteins identified from the OMVs enriched from mouse stools are listed in Supplemental Table S4.
